# Supplementary material for: Subfamily-specific differential contribution of individual monomers and the tether sequence to mouse L1 promoter activity
Source: Mob DNA. 2022 Apr 20;13:13. doi: 10.1186/s13100-022-00269-z (PMC9022269; doi:10.1186/s13100-022-00269-z)
Supplement: Supplementary file 2 — Additional file 2: F9 data, sequence alignments and cell line authentication report. Figure S1. Comparison of sense and antisense promoter activities for two-monomer mouse L1 5’UTR consensus sequences in F9 cells. Figure S2. Differential contribution of monomer 2, monomer 1 and tether to overall promoter activity in F9 cells. Figure S3. Contribution of different lengths of monomer 3 to overall promoter activity in F9 cells. Figure S4. Alignment of M2 from A_I, Gf_I and Tf_I subfamilies. Figure S5. Alignment of A_I monomers. Figure S6. Alignment of Tf_I monomers. Figure S7. Alignment of Gf_I monomers. Figure S8. Alignment of tether sequences. Figure S9. Cell line authentication report for NIH/3T3 subline used. [file 13100_2022_269_MOESM2_ESM.pdf]

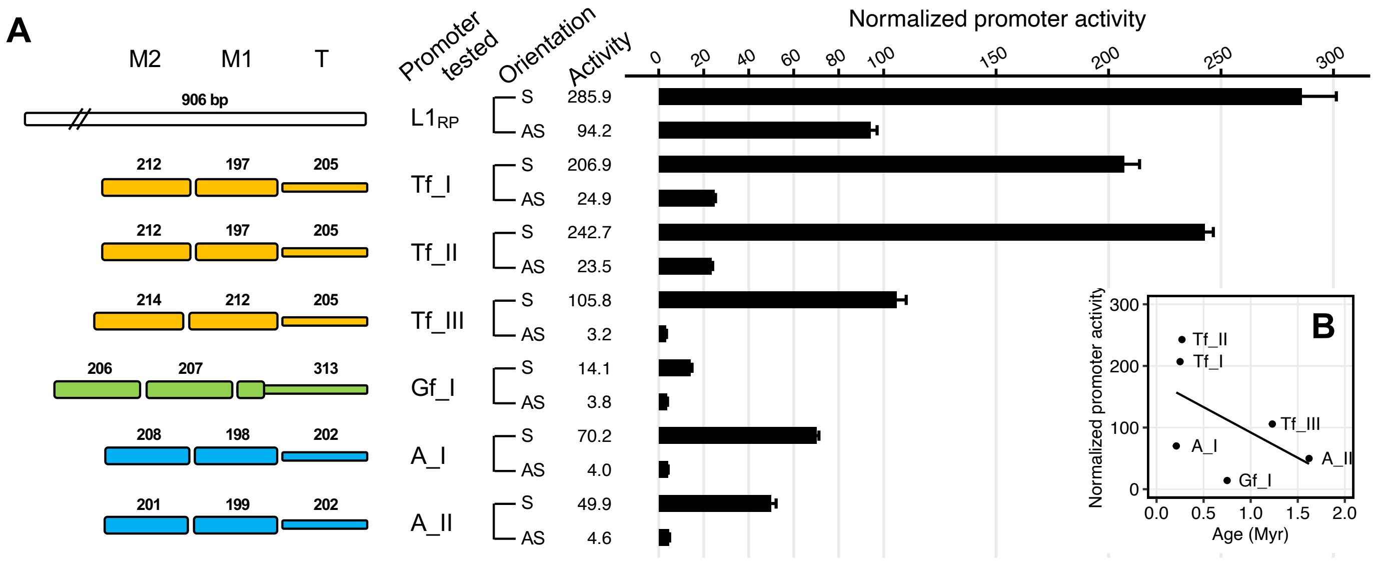

Figure S1. Comparison of sense and antisense promoter activities for two-monomer mouse L1 5'UTR consensus sequences in F9 cells. (**A**) Normalized activity of two-monomer consensus promoter sequences from six mouse L1 subfamilies. Sequence organization of the promoters is illustrated on the left side. The length of M2, M1, and tether (T) for each promoter is annotated (in base pairs). For each subfamily, the promoter activity was tested in both sense (S) and antisense (AS) orientation. The x-axis indicates the normalized promoter activity (i.e., the Fluc/Rluc ratio of a control no-promoter vector, pLK037, was set to 1). (**B**) Inverse relationship between the sense promoter activity and subfamily age. A simple linear regression line was shown along with individual data points (R = -0.57, p = 0.27).

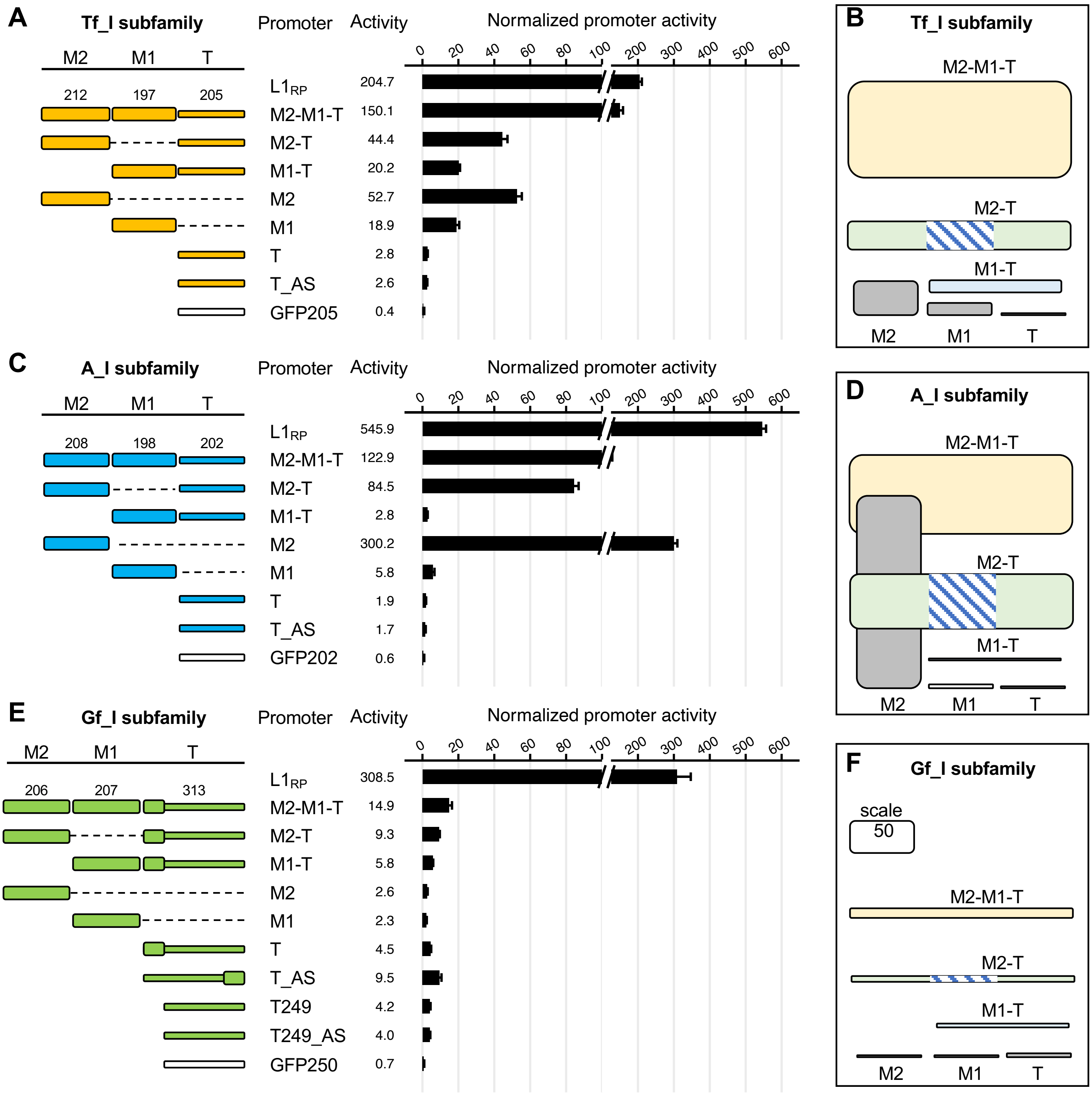

Figure S2. Differential contribution of monomer 2, monomer 1 and tether to overall promoter activity in F9 cells. Normalized promoter activity of individual 5'UTR domains for subfamily Tf_I (**A**), A_I (**C**), and Gf_I (**E**). Sequence organization of the promoters is illustrated on the left side. The length of M2, M1, and tether for each promoter is annotated (in base pairs). The dashed line represents domain(s) that were removed in reference to the two-monomer 5'UTR sequence (M2-M1-T). The tether was tested in both sense (T) and antisense (T_AS) orientation. A short version of Gf_I tether was additionally included (T249 and T249_AS) in panel E. The x-axis indicates the normalized promoter activity (i.e., the Fluc/Rluc ratio of a control no-promoter vector, pLK037, was set to 1). Note a broken x-axis was used to highlight the wide range of promoter activities. On the right hand are 2-D representations of the promoter data for subfamily Tf_I (**B**), A_I (**D**), and Gf_I (**F**), corresponding to panel A, panel C, and panel E, respectively. Each domain tested is represented by a filled box. The domains are arranged in the order of M2, M1, and Tether from left to right. The height of the box corresponds to the normalized promoter activity (to scale). A scale is shown in panel F; its height corresponds to a normalized promoter activity of 50. The hatched lines represent the missing M1 domain in the M2-T promoter construct.

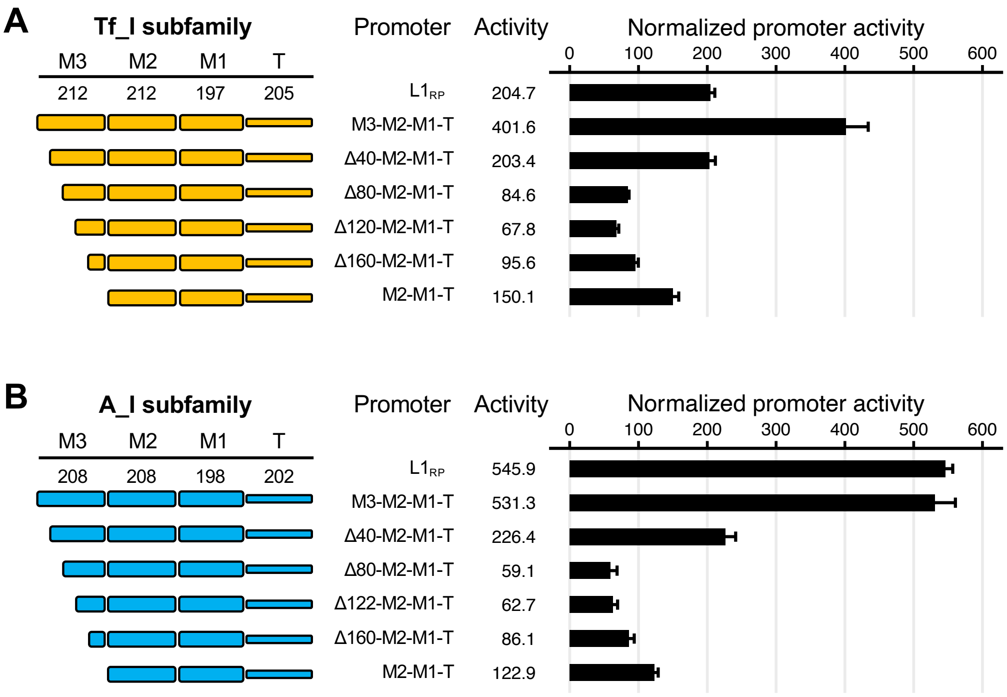

Figure S3. Contribution of different lengths of monomer 3 to overall promoter activity in F9 cells. (**A**) Normalized promoter activity of Tf_I 5'UTR consensus sequences with varying M3 length. Sequence organization of the promoters is illustrated on the left side. The length of M2, M1, and T for each promoter is annotated (in base pairs). The x-axis indicates the normalized promoter activity. Note promoter constructs in this panel were tested together in the same 96-well plate with those for Fig.S2A; thus, L1RP, M3-M2-M1-T and M2-M1-T are shared between Fig.S2A and Fig.S3A. (**B**) Normalized promoter activity of A_I 5'UTR consensus sequences with varying M3 length. Note promoter constructs in this panel were tested together with those for Fig.S2C.

Monomer 2 alignments

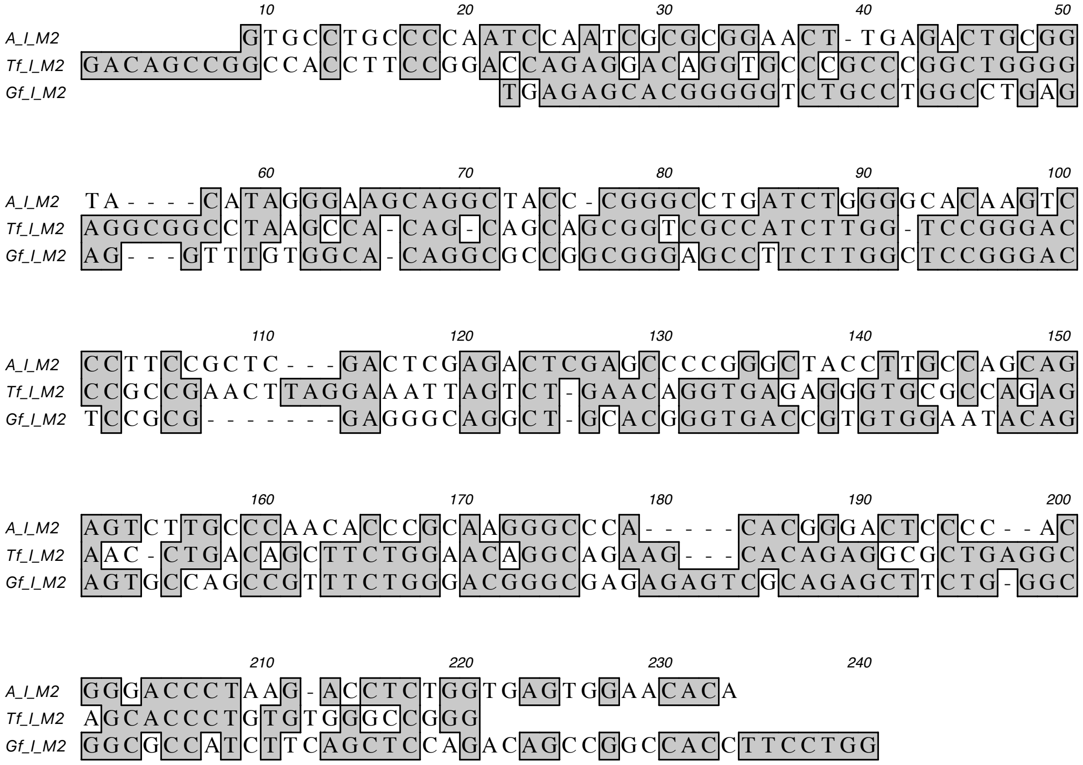

Figure S4. Alignment of M2 from A_I, Gf_I and Tf_I subfamilies.

Alignment of A_I monomers

```
                   10        20        30        40        50
A_Severynse1992    GTGCCTGCCCCAATCCAATCGCACGGAACTTGAGACTGCGGTACATAGGG
A-I_Schichman1993  GTGCCTGCCCCAATCCAATCGCGCGGAACTCGAGACTGCGGTACATAGGG
A1_Zhou2019        GTGCCTGCCCCAATCCAATCGCGCGGAACTTGAGACTGCGGTACATAGGG
A_I_M3             GTGCCTGCCCCAATCCAATCGCGCGGAACTTGAGACTGCGGTACATAGGG
A_I_M2             GTGCCTGCCCCAATCCAATCGCGCGGAACTTGAGACTGCGGTACATAGGG
A_I_M1             GCGCCTACCCCAATCCAATCGCGTGGAACTTGAGACTGCGGTACATAGGG

                   60        70        80        90        100
A_Severynse1992    AAGCAGGCTACCCGGGCCTGATCTGGGGCACAAGTCCCTTCCGCTCGACT
A-I_Schichman1993  AAGCAGGCTACCCGGGCCTGATCTGGGGCACAAGTCCCTTCCGCTCGACT
A1_Zhou2019        AAGCAGGCTACCCGGGCCTGATCTGGGGCACAAGTCCCTTCCGCTCGACT
A_I_M3             AAGCAGGCTACCCGGGCCTGATCTGGGGCACAAGTCCCTTCCGCTCGACT
A_I_M2             AAGCAGGCTACCCGGGCCTGATCTGGGGCACAAGTCCCTTCCGCTCGACT
A_I_M1             AAGCAGGCTACCCGGGCTTGATCTGGGGCACAAACCCCTTCCACTCCACT

                   110       120       130       140       150
A_Severynse1992    CGAGACTCGAGCCCCGGGCTACCTTGCCAGCAGAGTCTTGCCCAACACCC
A-I_Schichman1993  CGAGACTCGAGCCCCGGGCTACCTTGCCAGCAGAGTCTTGCCCAACACCC
A1_Zhou2019        CGAGACTCGAGCCCCGGGCTACCTTGCCAGCAGAGTCTTGCCCAACACCC
A_I_M3             CGAGACTCGAGCCCCGGGCTACCTTGCCAGCAGAGTCTTGCCCAACACCC
A_I_M2             CGAGACTCGAGCCCCGGGCTACCTTGCCAGCAGAGTCTTGCCCAACACCC
A_I_M1             CGAG-------CCCCGG-CTACCTTGCCAGCTGAGTC--GCCTGACACCC

                   160       170       180       190       200
A_Severynse1992    GCAAGGGTCCACACGGGACTCCCCACGGGACCCTAAGACCTCTGGTGAGT
A-I_Schichman1993  GCAAGGGTCCACACGGGACTCCCCACGGGACCCTAAGACCTCTGGTGAGT
A1_Zhou2019        GCAAGGGCCCACACGGGACTCCCCACGGGACCCTAAGACCTCTGGTGAGT
A_I_M3             GCAAGGGTCCACACGGGACTCCCCACGGGACCCTAAGACCTCTGGTGAGT
A_I_M2             GCAAGGGCCCACACGGGACTCCCCACGGGACCCTAAGACCTCTGGTGAGT
A_I_M1             GCAAGGGCCCACACAGGATTCCACACGTGATCCTAAGACCTCTAGTGAGT

A_Severynse1992    GGATCACA
A-I_Schichman1993  GGATCACA
A1_Zhou2019        GGAACACA
A_I_M3             GGATCACA
A_I_M2             GGAACACA
A_I_M1             GGAACACA
```

Figure S5. Alignment of A_I monomers.

Alignment of Tf_I monomers

```
                                 10        20        30        40        50
Tf_Naas1998          GACAGCCGGCCACCTTCCGGACCAGAGGACAGGTGCCCGCCCGGCTGGGG
Tf_DeBeradinis1999   GACAGCCGGCCACCTTCCGGACCGGAGGACAGGTGCCCGCCCGGCTGGGG
Tf_I_M3              GACAGCCGGCCACCTTCCGGACCGGAGGACAGGTGCCCGCCCGGCTGGGG
Tf_I_M2              GACAGCCGGCCACCTTCCGGACCAGAGGACAGGTGCCCGCCCGGCTGGGG
Tf_I_M1              GACAGCCGGCCACCTTCCGGACCAGAGGACAGGTGCCCGCCCGGCTGGGG

                                 60        70        80        90       100
Tf_Naas1998          AGGCGGCCTAAGCCACAGCAGCAGCGGTCGCCATCTTGGTCC-GGGAC-C
Tf_DeBeradinis1999   AGGCGGCCTAAGCCACAGCAGCAGCGGTCGCCATCTTGGTCC-GGGAC-C
Tf_I_M3              AGGCGACCTAAGCCACAGCAGCAGCGGTCGCCATCTTGGTCC-GGGAC-C
Tf_I_M2              AGGCGGCCTAAGCCACAGCAGCAGCGGTCGCCATCTTGGTCC-GGGAC-C
Tf_I_M1              AGGCGACCTAAGCCACAGCAGCAGCGGTCGCCATCTTGGTCCCGGGACTC

                                110       120       130       140       150
Tf_Naas1998          CGCCGAACTTAGGAAATTAGTCTGAACAGGTGAGAGGGTGCGCCAGAGAA
Tf_DeBeradinis1999   CGCCGAACTTAGGAAATTAGTCTGAACAGGTGAGAGGGTGCGCCAGAGAA
Tf_I_M3              CGCCGAACTTAGGAAATTAGTCTGAACAGGTGAGAGGGTGCGCCAGAGAA
Tf_I_M2              CGCCGAACTTAGGAAATTAGTCTGAACAGGTGAGAGGGTGCGCCAGAGAA
Tf_I_M1              CAAGGAACTTAGGAATTTAGTCTGCTTAGGTGAGAGTCTGTACCA-----

                                160       170       180       190       200
Tf_Naas1998          CCTGACAGCTTCTGGAACAGGCGGAAGCACAGAGGCGCTGAGGCAGCACC
Tf_DeBeradinis1999   CCTGACAGCTTCTGGAACAGGCGGAAGCACAGAGGCGCTGAGGCAGCACC
Tf_I_M3              CCTGACAGCTTCTGGAACAGGCGGAAGCACAGAGGCGCTGAGGCAGCACC
Tf_I_M2              CCTGACAGCTTCTGGAACAGGCAGAAGCACAGAGGCGCTGAGGCAGCACC
Tf_I_M1              CCTGGGAACTGCCAAAGCAA-------CACAGTGT--CTGAGAAAGGTCC

                                210
Tf_Naas1998          CTGTGTGGGCCGGG
Tf_DeBeradinis1999   CTGTGTGGGCCGGG
Tf_I_M3              CTGTGTGGGCCGGG
Tf_I_M2              CTGTGTGGGCCGGG
Tf_I_M1              TGTTTTGGGCC
```

Figure S6. Alignment of Tf_I monomers.

Alignment of Gf_I monomers

```
                                         10        20        30        40        50
Gf_Goodier2001  TGAGAGCACGGGGGTCTGCCNGGCCTGAGAGGTTTGTGGCACAGGCGCCG
Gf_I_M2         TGAGAGCACGGGGGTCTGCCTGGCCTGAGAGGTTTGTGGCACAGGCGCCG
Gf_I_M1         TGAGAGCACGGGGGTCTGCCTGGCCTGAGAGGTTTGTGGCACAGGCGCCG

                                         60        70        80        90       100
Gf_Goodier2001  GCGGGAGCCTTCTTGGCTCCGGGACTCCGCGGAGGGCAGGCTGCACGGGT
Gf_I_M2         GCGGGAGCCTTCTTGGCTCCGGGACTCCGCGGAGGGCAGGCTGCACGGGT
Gf_I_M1         GCGGGAGCCTTCTTGGCTCCGGGACTCCGCGGAGGGCAGGCTGCACGGGT

                                        110       120       130       140       150
Gf_Goodier2001  GACCGTGTGGAATACAGAGTGCCAGCCGTTTCTGGGACGGGCGAGAGNNN
Gf_I_M2         GACCGTGTGGAATACAGAGTGCCAGCCGTTTCTGGGACGGGCGAGAGAGT
Gf_I_M1         GACCGTGTGGAATACAGAGTGCCAGCCGTTTCTGGGACGGGCGAGAGAGT

                                        160       170       180       190       200
Gf_Goodier2001  CAGAGNNCTTCTGGG-CGGCGCCATCTTCAGCTCCAGACAGCCGGCCACC
Gf_I_M2         CGCAGAGCTTCTGGG-CGGCGCCATCTTCAGCTCCAGACAGCCGGCCACC
Gf_I_M1         CGCAGAGATTCTGGGGCGGCACCATCCTCAGCTCCAGACAACCAGCCACC

Gf_Goodier2001  TTCCTGG
Gf_I_M2         TTCCTGG
Gf_I_M1         TTCCCGG
```

Figure S7. Alignment of Gf_I monomers.

Alignment of tethers

```
                                                   10        20        30        40        50
A_I_tether
Tf_I_tether
Gf_I_tether  CAAGAGCCACAGAGCTTCTGAGGCTGCGACATCTTCGACTCCAGACAACT
Gf_I_T249

                                                   60        70        80        90       100
A_I_tether   --------------------------------------------------
Tf_I_tether  --------------------------------------------------
Gf_I_tether  GGCCACCTTCCTGGCCAAAGCAACACAGCTTCTGGGAAAGATCCTGTTTT
Gf_I_T249    --------------CCAAAGCAACACAGCTTCTGGGAAAGATCCTGTTTT

                                                  110       120       130       140       150
A_I_tether   ---------ACTTCTGCCAGGAGTCTGGTTCGAACACCAGATATCTGGGT
Tf_I_tether  -----TTCTTCTTCGGCCAGGAGGA-GGTCCAAATACAAGATATCTGCGC
Gf_I_tether  GGGCCTTCACCTTCAGCCAGGAGGA-GGTCCAAACACCAGATAACTGTAC
Gf_I_T249    GGGCCTTCACCTTCAGCCAGGAGGA-GGTCCAAACACCAGATAACTGTAC

                                                  160       170       180       190       200
A_I_tether   ACCTGCCTTGCAAGAAGAGAGCTTGCCTGCAGAGAATACTCTGCCCACTG
Tf_I_tether  ACCTTCCCTGTAAGAG---AGCTTGCCAGCAGAGAGTGCTCTGAGCACTG
Gf_I_tether  ACCTTCCCTGAGAGAGGAGAGCTTGCCTACAGAGACTGCTCTGACCACTG
Gf_I_T249    ACCTTCCCTGAGAGAGGAGAGCTTGCCTACAGAGACTGCTCTGACCACTG

                                                  210       220       230       240       250
A_I_tether   AAACT--AAGGAGAGTGC-TACCCTCCAGGTCTGCTCATAGAGGCTAACA
Tf_I_tether  AAACTCAGAGGAGAGAATCTGTCTCCCAGGTCTGCTGATAGACGGTAACA
Gf_I_tether  AAACTCAGAGAAGAGAGCTTGTCTCCCACGCCTGCTGATAGAGGGTAACA
Gf_I_T249    AAACTCAGAGAAGAGAGCTTGTCTCCCACGCCTGCTGATAGAGGGTAACA

                                                  260       270       280       290       300
A_I_tether   GAGTCACCTGAAGAACAAGCTCTTAACAGTGACAACTAAAACAGCTAGCT
Tf_I_tether  GAATCACCAGAAGAACAATCTCTAAACAGAGTCAACTATAACTACTAACT
Gf_I_tether  AAATCAACAGAGGAACAATCTTTTAACAAAGACAACTATAACAACTAACT
Gf_I_T249    AAATCAACAGAGGAACAATCTTTTAACAAAGACAACTATAACAACTAACT

                                                  310
A_I_tether   TCAGAGATTACCAG
Tf_I_tether  CCAGAGATTACCAG
Gf_I_tether  CCAGAGATTGCCAG
Gf_I_T249    CCAGAGATTGCCAG
```

Figure S8. Alignment of tether sequences.

Cell Line Authentication Service
Mouse STR Profile Report

ATCC

FTA Barcode: MUSA1026

ATCC Sales Order: SO0814026

Test Results for Submitted Sample					ATCC Reference Database Profile			
**Locus**	**Query Profile: MUSA1026**				**Database Profile: CRL-1658 NIH/3T3; Embryonic Fibroblast; Mouse**			
**18-3**	17	19			17	19		
**4-2**	19.3	20.3			19.3	20.3		
**6-7**	12				12			
**19-2**	11	12			11	12		
**1-2**	13	17			13	17		
**7-1**	29				29			
**1-1**	10				10			
**3-2**	14	15			14	15		
**8-1**	15				15			
**2-1**	9				9			
**15-3**	20.3				20.3			
**6-4**	15.3	16.3			15.3			
**11-2**	15	17			15	17		
**17-2**	13	14			13	14		
**12-1**	20				20			
**5-5**	14	15			14	15		
**X-1**	25				25			
**13-1**	16.2				16.2			
Number of shared alleles between query sample and database profile:								26
Total number of alleles in the query sample profile:								27
Total number of alleles in the database profile:								26
Percent match between the submitted sample and the database profile:								98

Explanation of Test Results
Cell lines with ≥80% match are considered to be related; i.e., derived from a common ancestry. Cell lines with a percent match between a 55 - 80% require further investigation for authentication of relatedness.

☐ The submitted sample profile is an exact match for the following ATCC cell line(s) in the ATCC mouse STR database:

☐ The submitted sample profile is mouse, however a matching reference profile has not previously been established in the ATCC mouse STR database.

☑ The submitted profile is similar to the following ATCC cell line(s): CRL-1658

☐ An STR profile could not be generated from the submitted sample.

Human and/or African Green Monkey Species Detection

☐ Human and/or African green monkey has been detected in the submitted sample profile (see attached electropherogram at Human D8 & D4 loci).

Additional Comments:

The submitted sample MUSA1026 (NIH/3T3) is similar to ATCC cell line CRL-1658 (NIH/3T3).

Figure S9. Cell line authentication report for NIH/3T3 subline used.
